# Multimodal characterization of the semantic N400 response within a rapid evaluation brain vital sign framework

**DOI:** 10.1186/s12967-018-1527-2

**Published:** 2018-06-04

**Authors:** Sujoy Ghosh Hajra, Careesa C. Liu, Xiaowei Song, Shaun D. Fickling, Teresa P. L. Cheung, Ryan C. N. D’Arcy

**Affiliations:** 1 Faculty of Applied Science, Simon Fraser University, Burnaby, BC Canada; 2 Health Science and Innovation, Surrey Memorial Hospital, Fraser Health Authority, Surrey, BC Canada; 3 HealthTech Connex Inc, Surrey, BC Canada; 4 Surrey NeuroTech Lab, Surrey Memorial Hospital, 13750 96 Avenue, Surrey, BC V3V 1Z2 Canada; 5 ImageTech Lab, Surrey Memorial Hospital, 13750 96 Av, Surrey, BC V3V 1Z2 Canada

**Keywords:** N400, ERP, MEG, Semantic language, Clinical application

## Abstract

**Background:**

For nearly four decades, the N400 has been an important brainwave marker of semantic processing. It can be recorded non-invasively from the scalp using electrical and/or magnetic sensors, but largely within the restricted domain of research laboratories specialized to run specific N400 experiments. However, there is increasing evidence of significant clinical utility for the N400 in neurological evaluation, particularly at the individual level. To enable clinical applications, we recently reported a rapid evaluation framework known as “brain vital signs” that successfully incorporated the N400 response as one of the core components for cognitive function evaluation. The current study characterized the rapidly evoked N400 response to demonstrate that it shares consistent features with traditional N400 responses acquired in research laboratory settings—thereby enabling its translation into brain vital signs applications.

**Methods:**

Data were collected from 17 healthy individuals using magnetoencephalography (MEG) and electroencephalography (EEG), with analysis of sensor-level effects as well as evaluation of brain sources. Individual-level N400 responses were classified using machine learning to determine the percentage of participants in whom the response was successfully detected.

**Results:**

The N400 response was observed in both M/EEG modalities showing significant differences to incongruent versus congruent condition in the expected time range (p < 0.05). Also as expected, N400-related brain activity was observed in the temporal and inferior frontal cortical regions, with typical left-hemispheric asymmetry. Classification robustly confirmed the N400 effect at the individual level with high accuracy (89%), sensitivity (0.88) and specificity (0.90).

**Conclusion:**

The brain vital sign N400 characteristics were highly consistent with features of the previously reported N400 responses acquired using traditional laboratory-based experiments. These results provide important evidence supporting clinical translation of the rapidly acquired N400 response as a potential tool for assessments of higher cognitive functions.

## Background

Measurements of brainwave activity through event-related potentials (ERPs) are becoming increasingly useful in providing objective, physiology-based measures of brain function [1]. ERPs are derived from electroencephalography (EEG), and can provide information about cortical electrical activity corresponding to different aspects of neural processing [2, 3]. In particular, higher order cognitive functions like semantic processing indexed by the N400 ERP are among the most promising responses for emerging clinical applications [4–7]. The N400 response was first described when Kutas and Hillyard presented participants with visual sentences that either had a semantically related (i.e. *congruent*) or semantically unrelated (i.e. *incongruent*) ending [8]. It was observed as a negative deflection of the incongruent relative to congruent condition waveforms which peaked at approximately 400 ms latency following stimulus presentation, and the authors suggested that this differential was a neural marker of semantic language processing.

In the 38 years since its initial report, the N400 response has been studied extensively using a variety of stimulus paradigms in various healthy and clinical populations [9–14]. While the initial N400 work utilized sentence-based stimuli, subsequent studies showed that prime-target word pairs also successfully elicited this response [15, 16]. Additionally, non-language-based stimuli such as mental arithmetic and action sequences have also been shown to produce the N400 response [17], and the strength of this response has been found to be correlated with various stimulus properties [18]. Others have demonstrated overlapping features in the temporal and spatial characteristics of the N400 response when elicited using language- as well as non-language-based stimuli [17], with the spectral content in particular demonstrating potential in distinguishing between different neural processes [19]. In fact, one of the key spectral features of the N400 response has been shown to be a reduction in beta band oscillations when processing incongruent relative to congruent stimuli in semantic language paradigms [20].

The cortical generators of the N400 response have been investigated using numerous noninvasive imaging modalities, such as functional magnetic resonance imaging (fMRI), electroencephalography (EEG), as well as magnetoencephalography (MEG). Results have revealed widespread cortical activations across the left temporal lobe, along with smaller areas of activity in the right temporal as well as bilateral inferior frontal and parietal regions [11, 21–23]. Specifically, areas of the bilateral temporal cortices (Brodmann Areas [BA] 20/21/22) and left inferior frontal gyrus (BA 45/47) have been shown to be key cortical regions within the distributed language network likely responsible for N400 [24], and these results are also supported by findings from lesion studies [25].

Further to its functional relevance as an indicator of neural processing in healthy individuals, the N400 response has also shown significant potential as a diagnostic and prognostic tool in clinical populations [4, 17, 26–33]. Studies in brain-injured patients with disorders of consciousness showed that the N400 response was correlated with functional recovery [4]. Moreover, changes in N400 response also predicted cognitive decline in patients as they progressed from mild cognitive impairment (MCI) to dementia [5, 31]. Yet despite these promising findings, the use of the N400 ERP beyond the research setting has been hindered by two main challenges: First, given that ERPs are produced by averaging the neural response signals across a large number of trials, traditional N400 studies require prolonged testing paradigms [1, 34]. These paradigms are particularly problematic in clinical populations due to fluctuations in vigilance levels and lack of capability or motivation [30, 35]. In addition, rather than measuring only a single brain response in clinical populations (e.g. sensation, attention, or language), there are now calls for concurrent evaluations of a *spectrum* of brain responses which provide a more complete profile of brain function [34]. This is particularly crucial in longitudinal monitoring of brain function changes in clinical populations [36]. Under these circumstances, the traditional ERP testing paradigms may require hours to evaluate, which is impractical within most clinical settings.

To assess the N400 response within a short testing time while providing information about other brain function indicators, our group has been undertaking systematic development of rapid evaluation techniques in recent years. We previously demonstrated the successful evaluation of the N400 response in 100 healthy individuals using a point-of-care enabled device [34], then employed this device to track the progress of rehabilitation therapy in a brain-injured patient [6]. More recently, we demonstrated a rapid evaluation platform known as the ‘brain vital sign’ framework [37], which enables the rapid assessment of several brain function indicators including the N400 (semantic language), N100 (sensory processing) [38] and P300 (attention orienting) [39]. The brain vital sign framework employs a portable, low-density EEG system, with automated, user-friendly software for easy clinical applications. The testing paradigm utilizes a short, 5-min auditory stimulus sequence in which tone and word stimuli are interlaced to maximize the number of trials and signal-to-noise ratio. Results in healthy adults showed that, not only were the target responses successfully elicited at the individual level, but the platform also captured expected age-related changes in attention and cognition that were undetected using conventional clinical screening measures [37].

Although the rapid evaluation brain vital sign framework showed initial promise as a potential avenue for clinical application of the N400 ERP, the component characteristics of this rapidly elicited N400 (rN400) response have not yet been described. Given the short, complex stimulus paradigm, it is crucial to characterize this response with respect to its spatiotemporal, spectral, and neuroanatomical features, and compare them with known N400 characteristics reported in studies using more conventional approaches over the last few decades.

The current study utilized MEG with simultaneous EEG to investigate the temporal, spatial, spectral, and neuroanatomical characteristics of the rN400 response elicited within the brain vital sign framework. We hypothesized that the rN400 response will exhibit features consistent with known characteristics of the N400 response, including: (1) increased ERP negativity and MEG signal power for the incongruent relative to congruent condition during the 300–500 ms post-stimulus interval; (2) decreased beta- band power for the incongruent relative to congruent condition during the same interval; and (3) increased activation of temporal and frontal cortices (BA 20, 21, 22, 45 and 47) for processing of incongruent relative to congruent stimuli.

## Methods

### Participant details

Seventeen (17) right-handed healthy participants with no history of neurological problems or psychoactive medication were recruited (22.6 ± 2.4 years, 10 males). Participants were undergraduate or graduate students, had normal hearing, normal or corrected-to-normal vision, and were fluent in English. The study was approved by ethics boards at Fraser Health Authority and Simon Fraser University, and all participants provided written informed consent.

### Auditory stimuli

**Fig. 1:**
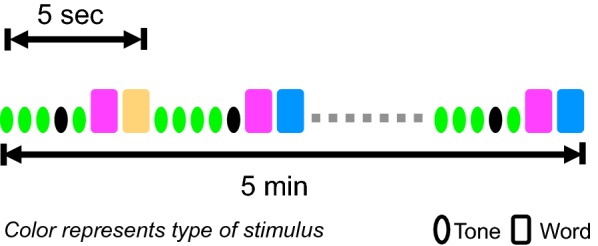
Illustration of auditory stimulus sequence of the brain vital sign framework. Blocks of five tones and two words repeated 60 times for a total scan time of about 5 min. Words represent prime-target pairs, containing both semantic congruent (pink–orange) and incongruent (pink–blue) pairs. Tones (standard = green and deviant = black) elicit sensory (N100) and attention (P300) measures

As introduced elsewhere [37], the rapid assessment framework utilizes a compressed auditory stimulus sequence with interlaced tones and words to elicit brain responses across four different functional domains—auditory sensation (N100 ERP), attention (P300 ERP), and semantic language (N400 ERP)—in approximately 5 min (Fig. 1). The sequence comprised 60 blocks, with each block containing five tones and two words representing a prime-target pair. Semantic language processing responses were derived from conditionally averaging the trials corresponding to the target word in the pair. Semantically linked words (*congruent* condition, 50%, e.g. doctor-nurse) were contrasted with words not semantically linked (*incongruent* condition, 50%, e.g. doctor-egg) to generate the differential processing measures. Words in both groups were balanced for characteristics such as word frequency and length, and the words in the semantically linked group had a minimum Cloze probability of 0.8 [40]. The stimuli were recorded in a male voice and root-mean-square normalized using Audacity software. The stimulus sequence contained 30 trials each of the congruent and incongruent conditions.

### MEG and EEG data acquisition

A 151-channel CTF MEG (MEG International Services Limited, Canada) was used with concurrent 3-channel EEG, both recorded in a magnetically shielded room with the participants in the supine position. Data were sampled at 1200 Hz using axial gradiometers (5-cm baseline) with synthetic 3rd order gradients employed for noise cancellation. Continuous head position monitoring was undertaken by three head position indicator coils located at fiducial points (HPI, positioned at nasion, left and right pre-auricular points). EEG recordings utilized Ag/AgCl scalp electrodes placed at Fz, Cz and Pz locations, with impedances kept below 5 kOhms. Four additional electrodes were placed on the head corresponding to reference (left mastoid), ground (forehead), horizontal (outer canthus of left eye) and vertical (supra-orbital ridge of left eye) electro-occulogram (EOG). To facilitate the alignment of MEG scanner and head coordinate systems, the shape of the participants’ head and the 3-dimensional position of HPI coils and EEG/EOG electrodes were recorded using a Polhemus electromagnetic digitization system prior to data collection (Polhemus Incorporated, USA). Auditory stimulation was presented binaurally using insert earphones, and participants were instructed to maintain visual fixation on a crosshair displayed on the overhead screen (white cross on black background) throughout the session.

### Data preprocessing

Raw data for both MEG and EEG were first visually inspected, and artifactual channels removed from further analysis. Data were then down-sampled to 300 Hz, notch filtered to remove frequencies corresponding to power line (60 Hz) with its harmonics as well as HPI coils, and low-pass filtered to 100 Hz. Data from 2 of the 17 participants were excluded from subsequent analysis due to poor quality.

### MEG analysis

Following band-pass filtering (0.5–45 Hz), independent component analysis (ICA) was performed with *runica* algorithm in EEGLAB [41] in order to remove artifact from ocular, cardiac, and muscular sources.

#### Temporal effects

Since head position within the MEG helmet can vary across participants, global field power (GFP) was utilized to provide a measure of the overall activity across all channels [42]. Individual-level GFP was computed for the congruent and incongruent conditions using trial-averaged event-related fields. A bootstrapping approach was utilized to determine time intervals of significant difference between conditions, in which the GFP signals at each time point were permuted between the congruent and incongruent conditions across all subjects [43]. Using this approach, the interval of significance was identified to be 300–500 ms and used as the window of interest in subsequent analyses, consistent with prior literature [44, 45]. The mean GFP value in this time interval was then calculated for each condition (congruent and incongruent) and participant, and compared using paired *t* test at the group level.

#### Spectral effects

Sensor level time–frequency analysis was undertaken by convolution of the data with Morlet wavelets (6 cycles) using the continuous wavelet transform function in MATLAB (The Mathworks Inc., USA). The coefficients corresponding to 0.5–45 Hz frequency in the − 200 to 900 ms time window relative to stimulus onset were extracted, and log power was computed as the square of the absolute value of the coefficients. To better understand the event-related spectral changes, the mean log power in the baseline period (− 100 to 0 ms) was subtracted from the log power in the post-stimulus period for every trial within the frequency band. Significance was assessed using a bootstrapping approach by permuting the trial-averaged wavelet power in the congruent and incongruent conditions across participants in each frequency [43]. This entailed the calculation of T-statistic for each time point and frequency between the congruent and incongruent conditions in the 800 ms following stimulus presentation. Thereafter, 1000 permutations were undertaken and new T-statistic calculated for every permutation leading to a null distribution against which the significance of the true T-statistic was assessed (with p < 0.05 considered to be significant).

#### Neuroanatomical effects

Source level analysis was performed using SPM8 (Welcome Trust Centre for Neuroimaging, UK) with the forward and inverse modeling steps elaborated in previously published work [46]. Source analysis for localizing neural generators of the semantic language process was undertaken using minimum norm estimates (MNE) to maintain consistency with prior N400 studies in MEG [24, 44]. Group constraints were employed during inversion [47], and source reconstruction was based on trial-averaged data within the entire frequency range (0.5–45 Hz) and active epoch (0–900 ms relative to stimulus presentation). Source-level contrast images were derived using data in the 0.5–45 Hz frequency range and previously identified window of 300–500 ms. Statistical modeling employed a general linear model (GLM) with T-contrasts [48].

### EEG analysis

To facilitate future translation into point-of-care enabled platforms, concurrently collected EEG data were also analyzed to extract ERPs. Contamination from ocular sources was removed from the EEG signal using an adaptive filtering approach [49]. For this process, the recorded EOG signals were used as reference inputs and processed using finite impulse response filters (m = 3), followed by recursive least squares-based removal from the EEG signal (λ = 0.9999). Subsequent to artifact removal, standard analysis steps including filtering (1–10 Hz), segmentation (− 200 to 900 ms) and conditional averaging were undertaken to generate ERPs [1, 2]. The mean value of the ERP waveform at the Cz electrode site in the 300–500 ms time interval was calculated for each condition and participant, and compared using paired *t* test at the group level.

#### Individual-level analysis

To evaluate reliability of the rN400 ERP at the individual level, a machine learning-based approach was undertaken using a two-category support vector machine (SVM) classifier following previously published methods [37, 50]. Briefly, an SVM classifier with a radial kernel was trained to distinguish between the congruent and incongruent condition waveforms using single-run, trial-averaged data from all three electrode sites. During each session, 90% of the available data were randomly selected to train the classifier, while the remaining 10% were used for testing classification accuracy. This procedure was repeated 10 times under tenfold cross-validation, such that the classifier was trained and tested on all available data. Results were averaged across all sessions, and measures were derived from the confusion matrix corresponding to accuracy, sensitivity, and specificity. To further assess the reliability of the analysis, results were verified using non-parametric permutation statistics [34, 51]. In short, this involved randomly redistributing the congruent and incongruent class labels among all datasets and performing the same classification procedures. This process was repeated 1000 times, and the resulting accuracies were used to create a null distribution against which the true classification accuracy was compared. Probabilities less than 0.05 were deemed to be significant for SVM classification outcome.

## Results

### Temporal and spectral effects in MEG

**Fig. 2:**
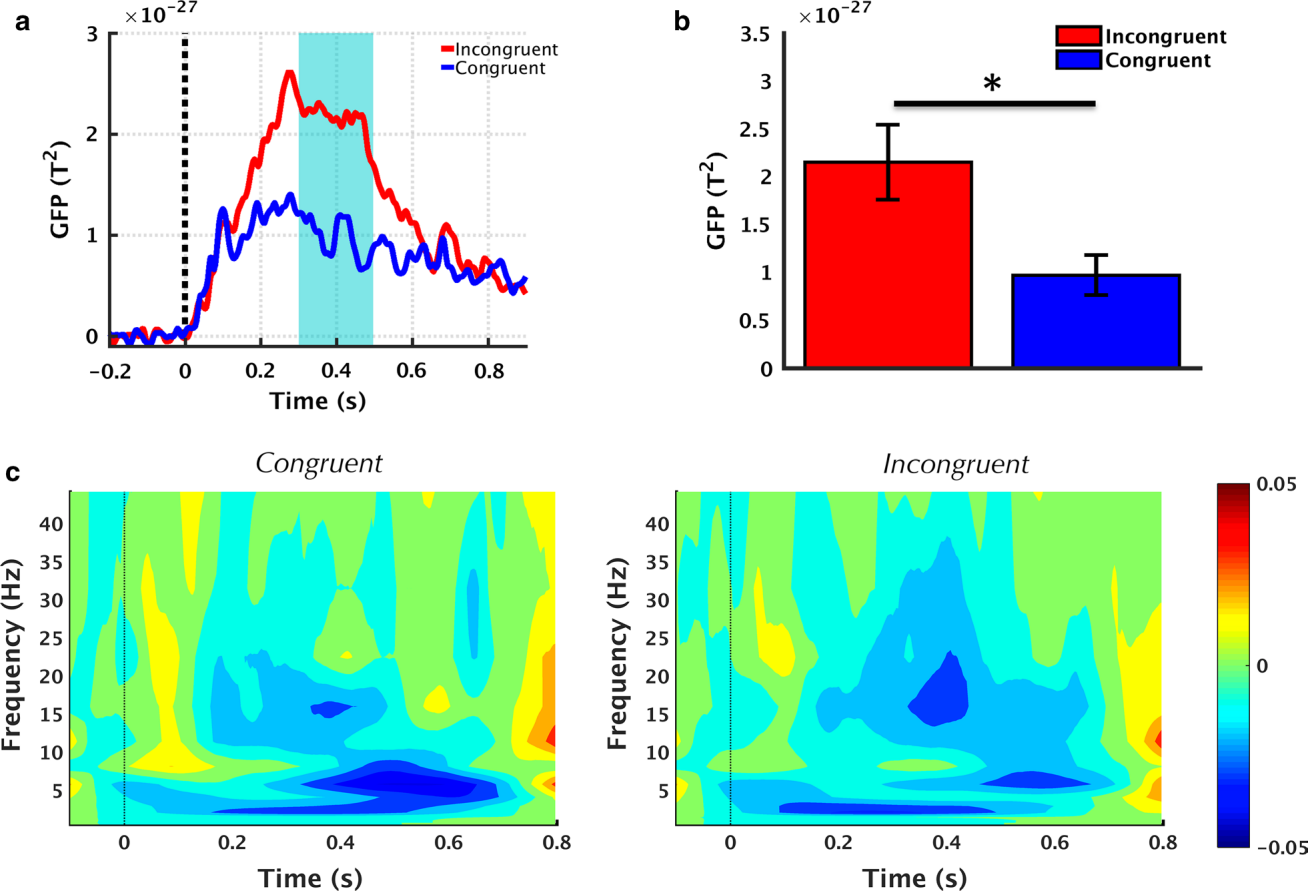
Sensor-level MEG results showing differential processing in incongruent compared to congruent condition. **a** Grand-averaged GFP demonstrating increased power for incongruent relative to congruent condition. Shaded region denotes window of interest (300–500 ms). **b** Mean GFP averaged across the time window specified in part A, calculated for each subject and presented as mean ± SEM across subjects. *p < *0.05.* **c** Time–frequency wavelet spectral power averaged over all MEG channels. Colour bar represents log power values

Sensor-level GFP demonstrated differential processing of the target word depending upon whether they were semantically related (*congruent* condition) or semantically unrelated (*incongruent* condition) to the first word. In particular, in the 300–500 ms post-stimulus interval, there was increased power for the incongruent relative to congruent condition (p < 0.05, Fig. 2a, b). In addition, the processing of incongruent words resulted in a significant reduction in beta band power relative to the processing of congruent words (p < 0.05, Fig. 2c). This decrease was observed in the 335–440 ms time interval, overlapping in time with the N400 response. Although there appeared to be some differences also present in other frequency bands, none of them were statistically significant.

### Temporal effects in EEG

**Fig. 3:**
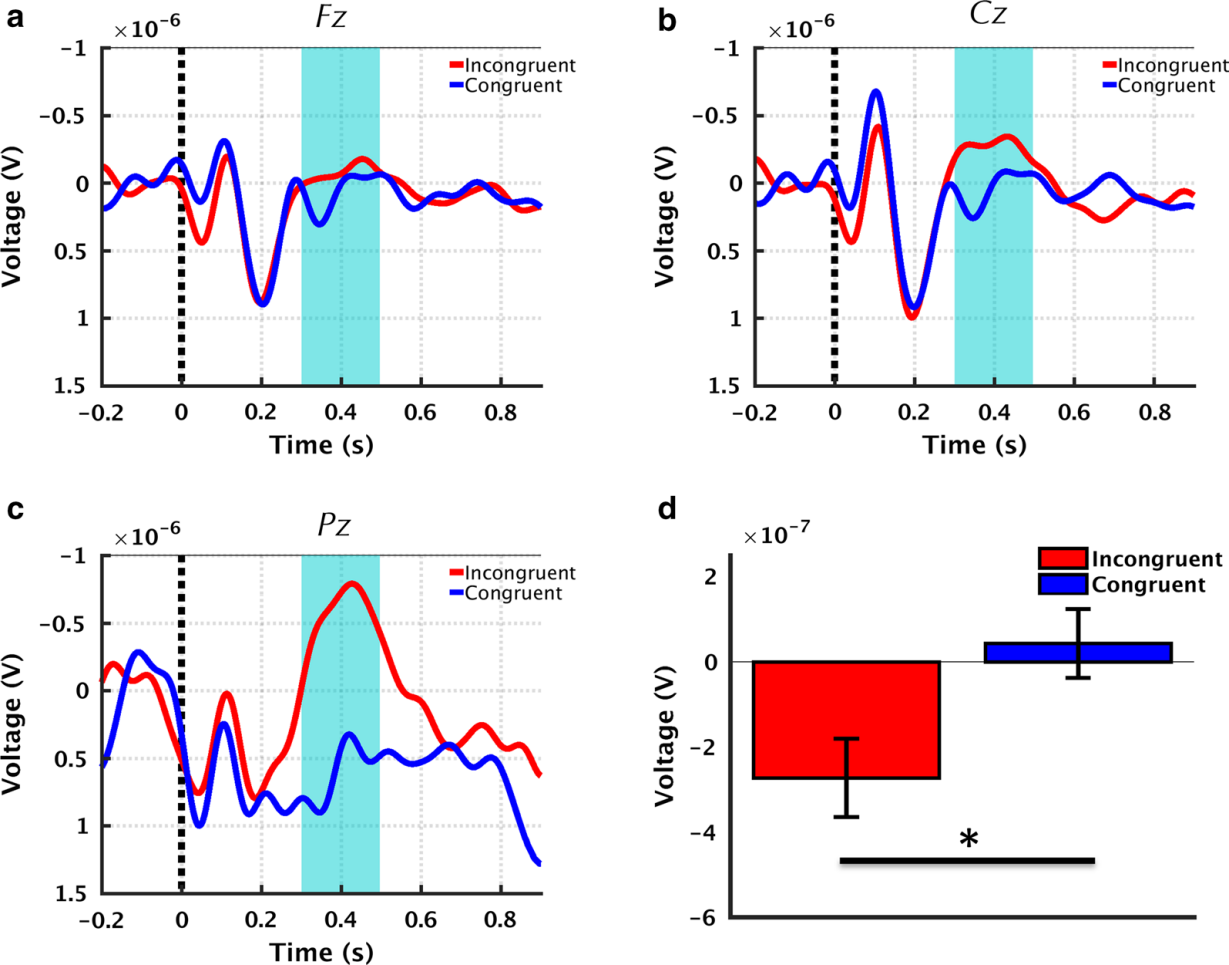
ERP results demonstrating differential processing of semantic congruence and incongruence. **a**–**c** Grand-averaged ERP waveforms at the Fz, Cz, and Pz electrode sites, respectively. Shaded regions denote windows of interest (300–500 ms). **d** Mean ERP amplitudes averaged over the windows of interest, calculated for each subject and presented as Mean ± SEM across subjects. *p < 0.05

ERP waveforms exhibited greater negativity in the incongruent relative to congruent condition occurring within the 300–500 ms interval, which was maximal at the Pz electrode (p < 0.05, Fig. 3a–c). The trained SVM classifier successfully distinguished between the congruent and incongruent conditions with 88.89% accuracy, 88% sensitivity, and 90% specificity. All classification results were verified to be statistically significant through permutation analysis (p < 0.05).

### Neuroanatomical effects in MEG

**Fig. 4:**
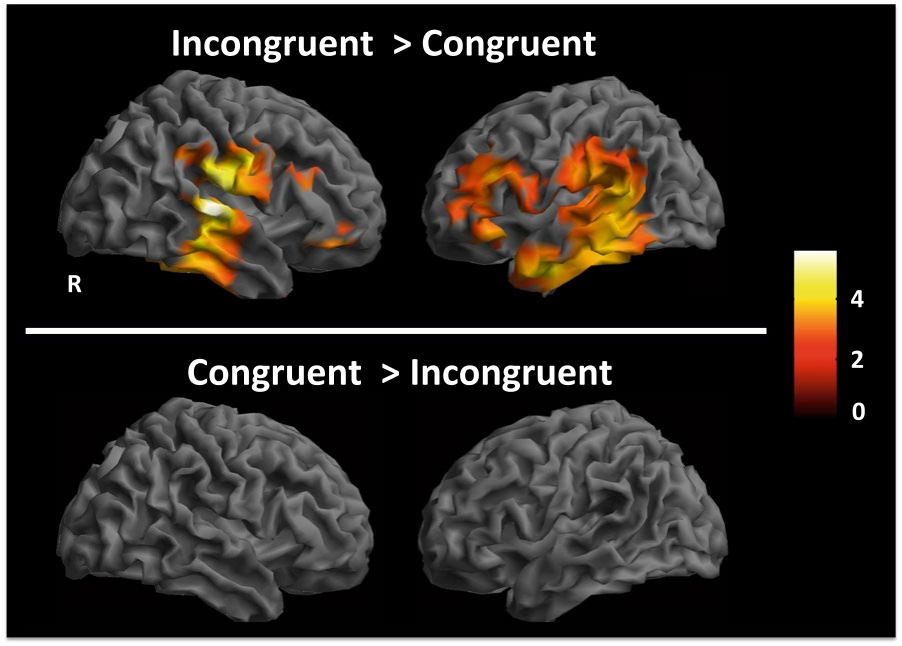
Source localization results. Top: Incongruent word processing activates a left-lateralized distributed region of cortex including temporal, inferior frontal and inferior parietal areas (incongruent > congruent contrast, p < 0.005unc.). Bottom: No suprathreshold clusters were identified for the reverse contrast (congruent > incongruent). Color bar represents T-statistic values

Differential processing of incongruent words was source-localized to the inferior frontal, inferior parietal, and temporal regions (incongruent > congruent contrast, p < 0.005, k = 20). Key areas included left inferior, middle and superior temporal gyri (BA 20, 21 and 22) and regions encompassing both the anterior and posterior portions of the left inferior frontal gyrus (BA 45, 47). Additionally, areas of the right temporal and inferior frontal gyri were also activated. In comparison, no suprathreshold clusters were observed for the reverse contrast of congruent > incongruent (Fig. 4 bottom panel).

## Discussion

### Main findings

This study employed MEG with concurrent EEG to investigate the temporal, spectral, and neuroanatomical characteristics of the rapidly elicited N400 response (rN400) generated through the brain vital sign framework. Using a compressed auditory stimulus sequence comprising both tones and prime-target word pairs, we demonstrated that the resulting rN400 response exhibited features consistent with characteristics previously reported for the N400 response in semantic language paradigms [17, 18]. In particular, we found that: (1) the sensor-level temporal characteristics showed rN400 ERP in the incongruent relative to congruent condition, peaking approximately 300–500 ms after stimulus presentation and with concomitant changes in GFP (Hypothesis 1); (2) a significant decrease in beta-band spectral power was observed during the same interval in the incongruent relative to congruent condition (Hypothesis 2); and (3) source localization analysis showed that rN400 processes activated cortical regions spanning the temporal, inferior frontal, and parietal regions known to be associated with the N400 response (Hypothesis 3). These main findings are summarized in Table 1.

**Table 1:** Comparison of the features of interest between the N400 response elicited using traditional approaches and the rN400 response elicited under the rapid assessment brain vital sign framework

Modality	Feature of interest	Traditional approach N400	Rapid framework (rN400)
EEG	Peak amplitude (cong. vs. incong.)	ERP: |V_incong_| > |V_cong_|^a^	ERP: |V_incong_| > |V_cong_|
	Peak latency (ms)	~ 400 ms^a^	420 ms
	Scalp topography	Centro-parietal maxima^b^	Max at parietal (Pz)
MEG	Amplitude difference (cong. vs. incong.)	∆_300–500 ms_^c,d^	∆_300–500 ms_
	Spectral effects	⇓ beta-band power^e^	⇓ beta-band power
	Cortical activation	⇑ IFG, TL, IPL^c,d,f^	⇑ IFG, TL, IPL

Effects are based on comparison of the incongruent condition with the congruent condition data. EEG-based features include peak amplitude (V), peak latency (ms), and scalp topography. MEG-based features include amplitude difference during the 300–500 ms window (∆_300–500ms_), spectral effects, and cortical activations. *Cong.*  congruent condition, *incong.*  incongruent condition, *IFG* inferior frontal gyrus, *TL* temporal lobe (superior, middle and inferior temporal gyri), *IPL* inferior parietal lobule. Only statistically significant features are shown

^a^ Kutas and Federmeier [17], ^b^ Lau et al. [9], ^c^ Halgren et al. [44], ^d^ Maess et al. [24], ^e^ Wang et al. [20], ^f^ Helenius et al. [23]

### Hypothesis 1: temporal effects

The sensor-level temporal effects showed a robust rN400 ERP for processing the incongruent relative to the congruent words (Fig. 3), consistent with previous findings based on sentences and semantic prime-target word pairs within auditory and visual modalities [17]. The response in the present study was observed to be maximal at the parietal (Pz) electrode location, also consistent with prior works suggesting a centro-parietal scalp distribution for the N400 ERP [9]. Importantly, these findings were also supported by our concurrent results using MEG which measures the magnetic counterpart of the rN400 ERP. Results showed that sensor-level GFP exhibited increased activity in the incongruent relative to congruent condition, peaking at similar latencies relative to rN400 ERP (Fig. 2a, b). It is important to note that polarity differences between the two modalities may be accounted for given that GFP is a power measure and is thus always non-negative, whereas ERP can be either positive or negative.

While the present study targeted the semantic processing effect indexed by the N400 and accordingly focused on the 300–500 ms window of interest to be concordant with previous literature [18, 44], other temporal differences between the two conditions were also present at earlier latencies within the ERP/ERF traces. These effects may be related to processes in support of semantic language comprehension such as phonological matching [52], letter-string processing [45] or detection of mismatch based on predicted input [53]. These earlier effects may be further explored in future studies.

### Hypothesis 2: spectral effects

Time–frequency results demonstrated a significant decrease in beta band power in the incongruent condition relative to the congruent (Fig. 2c). These spectral changes occurred over the same time interval as the rN400 response, and provide further confirmatory evidence of the processing differences between the two conditions. A previous MEG study reported similar beta-band power reductions, and source-localized this effect to the left inferior frontal gyrus and temporal regions, with the authors postulating that the observed N400 effects may have represented a dynamic communication link between these regions [20]. Additionally, beta band power suppression has also previously been associated with increased level of cortical processing across a diverse range of experimental paradigms, such as motor movement [54], working memory [55] and information retrieval [56]. In light of these findings, the reduction in beta band power observed in the current study may be interpreted as a potential reflection of increased processing for the incongruent relative to congruent conditions within the relevant brain regions. It should also be noted that, although reduced power is visually observed for the theta frequency band in the current study, this effect was not statistically significant.

### Hypothesis 3: neuroanatomical effects

Our results showed left-lateralized activations in the temporal cortices (BA 20, 21, 22) as well as inferior frontal gyri (BA 44, 45) (Fig. 4 upper panel). This is in agreement with prior works using fMRI and EEG, confirming the left temporal lobe as the largest source of the N400 effect, with a smaller contribution from the right temporal areas [21]. In addition, other EEG based works have identified contributions from the left perisylvian cortex [11], and bilateral inferior frontal gyri [22]. MEG based source localization has largely confirmed these findings, and suggested contributions from cortical areas including the left superior and middle temporal gyri as well as the inferior parietal and frontal areas [23, 44]. The converging neuroimaging results and theoretical models [9, 57, 58] have led to increasing consensus that semantic language processing is supported by a left lateralized network of brain regions [9, 24, 44]. Our results are consistent with these previous findings, as more left-lateralized activations were observed in both the temporal and inferior frontal regions. In addition to the left hemisphere activity, the right hemisphere activations observed in the current study were also in line with other studies using auditory stimuli [59].

The lack of suprathreshold clusters in the congruent > incongruent contrast (Fig. 4 lower panel) is also consistent with previous literature. MEG studies of N400 have shown largely overlapping areas of activation in both congruent and incongruent conditions, with greater extent of activations in the incongruent condition due to increased demands associated with incongruent stimulus [24]. Similarly, fMRI results showed increased hemodynamic activity for the incongruent condition compared to congruent [21]. Together, these hemodynamic and electromagnetic results support our findings regarding lack of suprathreshold clusters in the congruent > incongruent contrast.

### Clinical implications

Beyond the extensive laboratory based evaluations of N400, clinical applications are increasingly utilizing the N400 response in a variety of patient populations. The N400 is being particularly studied in disorders of consciousness (DOC) as a potential marker of residual functional integrity as well as for tracking rehabilitation progress. Beukema and colleagues reported the importance of including N400 in assessments of DOC patients [7], while Steppacher et al. demonstrated the N400 as a crucial tool for assessing information processing abilities that are predictive of eventual recovery in DOC patients [4]. Similarly, the N400 response has also been utilized to track rehabilitation progress in traumatic brain injury [6] and for assessments of stroke patients [28]. Moreover, the N400 response has been found to be abnormal in Alzheimer’s disease [60], and was identified as a promising marker in differentially identifying MCI patients who may transition to dementia [5]. These demonstrations in clinical populations, combined with the excellent reliability and stability of N400 effects [61] provide an impetus for clinical integration of this promising response. The present study makes N400 assessments clinically accessible by balancing the need for rapid assessments in clinical settings with the inherent desire for high quality data while retaining the key known features of the N400 response. Our results demonstrated that the rapidly elicited N400 response through the brain vital sign framework exhibit many of the similar characteristics compared to traditional N400 paradigms [9, 17, 62].

Additionally, the robust identification of the N400 effect at the individual level using automated expert-independent machine learning approaches provides additional support for clinical application of this rapid assessment technique. The 89% hit rate in the present study is quite comparable to previous reports—with prior machine learning based analysis reporting results in the 86–92% range [34, 37] and other analytical techniques also reporting observable N400 effects in similar proportions of healthy participants [7, 30].

### Caveats

Despite the promising findings in this study, two main limitations should also be noted. As this is the first study characterizing the rapidly elicited rN400 response within the brain vital sign framework, the focus was on examining its spatiotemporal and neuroanatomical effects and comparing them with known features of the traditional N400 response. However, given the myriad of language- and non-language-based experimental paradigms in which the N400 response has previously been described, it is not feasible to compare the rN400 response to every other traditional paradigm in one study. Rather, the current study focused on comparisons with language-based paradigms, and utilized response features and characteristics that have been identified as commonalities across different studies in order to account for variable modalities and experimental parameters (e.g. experimental condition, stimulus duration and type, inter-stimulus interval) [9, 17, 62]. Nonetheless, future studies may be conducted to examine more detailed comparisons between the brain vital sign rN400 response and traditional N400 responses. Additionally, as the first study of rN400 response, the current study utilized a distributed source modeling approach for source localization to be consistent with previous MEG studies of N400 [24, 44]. However, given the inherent limitations of this approach in biasing sources towards the cortical surface, future studies are needed to confirm these results using alternate source localization techniques such as spatial filtering using beamformer [63].

## Conclusion

In this study, we investigated the spatiotemporal and neuroanatomical features of the N400 response as elicited by the rapid assessment brain vital signs framework. Using both MEG and EEG, our results showed that the rapidly elicited N400 response exhibits characteristics consistent with those reported in traditional semantic language-based N400 paradigms. These characteristics include temporal features showing maximal response within 300–500 ms latency; topographic scalp distribution demonstrating maximal response at the posterior Pz electrode; spectral effects showing reduction in beta band power; and source localization to left-lateralized temporal and inferior frontal areas. With the increasing use of the N400 response in patient assessments for neurological conditions such as dementia and traumatic brain injury, the convergent M/EEG results of the current study provide further support for the possibility of translating the N400 response from research to clinical settings through a rapid assessment framework for evaluating cognitive functions.

## Abbreviations

ERP: event related potential
MEG: magnetoencephalography
BA: brodmann area
rN400: rapidly elicited N400
GFP: global field power
GLM: general linear model
IFG: inferior frontal gyrus
TL: temporal lobe
EEG: electroencephalography
fMRI: functional magnetic resonance imaging
MCI: mild cognitive impairment
ICA: independent component analysis
MNE: minimum norm estimate
SVM: support vector machine
IPL: inferior parietal lobule
DOC: disorders of consciousness
